# Fibrinogen Aα Thr312Ala Polymorphism Specifically Contributes to Chronic Thromboembolic Pulmonary Hypertension by Increasing Fibrin Resistance

**DOI:** 10.1371/journal.pone.0069635

**Published:** 2013-07-22

**Authors:** Ji-Feng Li, Yuan Lin, Yuan-Hua Yang, Hui-Li Gan, Yan Liang, Jie Liu, Su-Qiao Yang, Wei-Juan Zhang, Na Cui, Lan Zhao, Zhen-Guo Zhai, Jun Wang, Chen Wang

**Affiliations:** 1 Department of Respiratory and Critical Care Medicine, Beijing Chao-Yang Hospital, Capital Medical University, Beijing, P.R. China; 2 Beijing Key Laboratory of Respiratory and Pulmonary Circulation Disorders, Beijing Institute of Respiratory Medicine, Beijing Chao-Yang Hospital, Capital Medical University, Beijing, P.R. China; 3 Department of Physiology, Capital Medical University, Beijing, P.R. China; 4 Department of Respiratory Disease, Capital Medical University, Beijing, P.R. China; 5 Department of Cardiac Surgery, Beijing An-Zhen Hospital, Capital Medical University, Beijing, P.R. China; 6 Centre for Pharmacology and Therapeutics, Experimental Medicine, Imperial College London, London, United Kingdom; Ottawa Hospital Research Institute, Canada

## Abstract

**Background:**

Polymorphisms are associated with chronic thromboembolic pulmonary hypertension (CTEPH) and pulmonary thromboembolism (PTE), but no polymorphism specific to CTEPH but not PTE has yet been reported. Fibrin resistance is associated with CTEPH, but the mechanism has not been elucidated.

**Methods:**

Polymorphisms were analyzed in 101 CTEPH subjects, 102 PTE subjects and 108 healthy controls by Massarray or restriction fragment length polymorphism (RFLP). Plasmin-mediated cleavage of fibrin was characterized in 69 subjects (29 with CTEPH, 21 with PTE and 19 controls).

**Results:**

Genotype frequencies and allele frequencies of fibrinogen Aα Thr312Ala were significantly higher in CTEPH subjects than in controls and PTE subjects, while there was no difference between PTE subjects and controls. The odd ratio (OR 2.037) and 95% confidence interval (95% CI, 1.262–3.289) showed that Thr312Ala polymorphism was a risk factor for CTEPH but not PTE. Fibrin from CTEPH subjects was more resistant to lysis than that from PTE subjects and controls. Fibrin resistance was significantly different between Aα Thr312Ala (A/G) genotypes within CTEPH subjects, and the fibrin with GG genotype was more resistant than that with AA and AG genotype.

**Conclusions:**

Fibrinogen Aα Thr312Ala (A/G) polymorphism was associated with CTEPH, but not PTE, suggesting that the fibrinogen Aα Thr312Ala polymorphism may act as a potential biomarker in identifying CTEPH from PTE. GG genotype polymorphism contributes to CTEPH through increasing fibrin resistance, implying that PTE subjects with fibrinogen Aα GG genotype may need long-term anticoagulation therapy.

## Introduction

Chronic thromboembolic pulmonary hypertension (CTEPH), characterized by chronic and organized thromboembolic obstruction of the main, lobar and/or segmental pulmonary arteries, is a significant health threat [1], [2], [3]. While it is currently hypothesized that CTEPH is caused by unresolved thromboembolus of acute pulmonary thromboembolism (PTE) and increase in pulmonary vascular resistance (PVR), the pathogenesis of CTEPH remains unclear [3], [4]. In clinical practice, thromboembolus in most PTE subjects is dissolved within several weeks to several months after treatments such as fibrinolysis [5], however, a minority of patients failed to properly resolve thromboembolus [6]. Evidence suggests that CTEPH is triggered by failure to dissolve PTE [7], [8].

Fibrin derived from CTEPH subjects is more resistant to lysis by proteases (plasmin) than that derived from healthy controls in *in vitro* experiments [9]. Interestingly, fibrin resistance was also reported in subjects with pulmonary hypertension (PH) other than thromboembolic [10]. Minor resistance of fibrin was also observed in subjects with prior pulmonary embolism and no evidence of pulmonary hypertension [10]. These data indicate that fibrin resistance may play an important role in the development of CTEPH.

Fibrin resistance may be affected by factors such as fibrin stabilization or fibrinolysis. Fibrinogen polymorphisms are associated with thrombotic diseases such as myocardial infarction and stroke [11]. Among most common inherited thrombotic risk factor abnormalities, such as antithrombin, protein C, protein S, factor V and factor II mutations [12], there has been no frequency difference to date between CTEPH and PTE. Recently fibrinogen Aα Thr312Ala polymorphism was shown to be related with CTEPH [13] and PTE [14], and interestingly, Thr312Ala polymorphism was associated with thicker fibrin fibers and more extensive alpha-chain cross-linking [15].

In the present study, we screened the seven reported polymorphisms associated with CTEPH [13] from human subjects to determine which polymorphism correlates with CTEPH by influencing fibrin resistance.

## Materials and Methods

### Subjects

Between November 2006 and November 2010, 101 subjects with CTEPH were analyzed. Between January 2008 and June 2010, 102 PTE subjects were analyzed. Of the PTE subjects, 58 were without evidence of chronic pulmonary hypertension at the time of sampling and 44 were without pulmonary hypertension by two years follow-up. 108 healthy controls (19 subjects with well-controlled hypertension) with no history or evidence of venous thromboembolism were also analyzed. All subjects were Han Chinese and from Beijing Chao-Yang Hospital affiliated with the Capital Medical University. The Ethics Committee of Beijing Chao-Yang Hospital of Capital Medical University approved all experimental procedures. An informed consent form was provided according to the Declaration of Helsinki, and written informed consent was obtained according to institutional guidelines from all subjects. All subjects were evaluated according to a standardized protocol, including clinical history, physical examination, laboratory tests and computed tomography of pulmonary angiography (CTPA) or magnetic resonance pulmonary angiography (MRPA) or ventilation/perfusion scan (V/Q scan), transthoracic echocardiography, ultrasound check of lower extremity when PTE or CTEPH was suspected and right heart catheterization as pulmonary hypertension was suspected. CTEPH and PTE were diagnosed according to the corresponding guidelines. All PTE subjects had received standard therapy for PTE consisting of one-week unfractionated or low molecular weight heparin followed by oral anticoagulation for at least six months.

### Blood Sampling and Processing

Blood samples (2 ml) were drawn from veins, collected in Vacutainer tubes containing sodium citrate and centrifugated immediately at 3000 rpm for 15 minutes at room temperature. 0.5 ml aliquots of plasma and blood cells were stored at −80°C for further analysis.

### Genomic DNA Extraction and Genotyping

Genomic DNA was extracted from blood cells by a genomic DNA extraction kit from TIANGEN BIOTECH CO. LTD (Beijing, China, catalog NO. DP304). Polymorphisms were detected by MassArray (Sequenom, Bioyong Technologies Inc, Beijing, China) or restriction fragment length polymorphism (RFLP). Seven single nucleotide polymorphisms (SNPs) including fibrinogen Aα Thr312Ala (rs6050), fibrinogen Bβ Arg448Lys (rs420), fibrinogen Bβ −148C/T (rs1800787), fibrinogen Bβ −455G/A (rs1800790), prothrombin 19911A/G (rs3136516), plasminogen activator inhibitor-1 (PAI-1 4G/5G, rs1799768), tissue plasminogen activator 7351C/T (t-PA, rs2020918) were studied. Fibrinogen Aα Thr312Ala (rs6050), fibrinogen Bβ 148C/T (rs1800787) and fibrinogen Bβ 455G/A (rs1800790) SNPs were detected by RFLP, other polymorphisms were detected by MassArray.

### Fibrinogen Purification

Fibrinogen was purified using ethanol precipitation (details in Protocol S1). The purity of the fibrinogen was assessed by densitometry of Coomassie stained polyacrylamide gels after electrophoresis under reducing conditions. The concentration of the fibrinogen was determined by ultraviolet spectroscopy.

### Fibrin Formation and Digestion with Plasmin

Fibrin clots were prepared in microcentrifuge tubes by incubating thrombin (2 units/ml final concentration) with fibrinogen (2 mg/ml final concentration) in 25 µl of forming buffer [50 mM Tris (pH 7.4), 100 mM NaCl, and 20 mM ethylenediaminetetraacetic acid] for 1 h at room temperature. Plasmin [P1867, Sigma-Aldrich (Shanghai) Trading Co. Ltd] was then added (50 µl of 25 µg/ml) for clot digestion for various time periods (0 h, 1 h, 3 h, 6 h and 12 h) at 37°C. The digestion reaction was then terminated and samples heated at 95°C for 5 minutes and stored at −20°C.

### Electrophoretic Analysis of Fibrin

Aliquots of plasmin-digested samples (equivalent to 5 µg of fibrin) were assessed using sodium dodecyl sulfate-polyacrylamide gel electrophoresis (SDS-PAGE) (10% polyacrylamide gels). Band intensities of Coomassie-stained gels were scanned and analyzed by Image J software (Version 2.1). Data were normalized to the band intensity of the undigested control (0 h).

### Statistical Analysis

Genotype and allele frequencies were calculated for each locus. Observed frequencies in all groups were compared with those predicted by the Hardy-Weinberg equilibrium equation using the Chi-squared test. Any SNP deviated significantly from Hardy-Weinberg equilibrium was excluded for statistic analysis. Genotype and allele frequencies were analyzed using the Chi-squared test, or Fisher’s exact test in the case of low numbers. The risk of disease associated with each genotype was estimated by logistic regression analysis, where SNP genotype was coded in a three-level fashion, i.e. most rare homozygous genotype, heterozygous genotype and most common homozygous genotype, with the latter being the reference category. All analyses were repeated using multiple logistic regression models that adjusted for any index within the basic demographic or risk factors with differences between groups. *P* value of less than 0.05 was considered statistically significant and *P* value of less than 0.01 was considered highly significant. All statistics were performed using SPSS 13.0 software.

## Results

### Clinical Data of Subjects

Polymorphisms are associated with CTEPH or PTE. Therefore, CTEPH and PTE patients were analyzed in the present investigation. 101 CTEPH subjects, 102 PTE subjects and 108 healthy controls were analyzed and the clinical data are shown in Table 1. The average age at first diagnosis was similar in the three groups, but the male/female ratio was higher in CTEPH group (62%). Percentage of subjects with smoking history was similar in three groups, but the percentages of subjects with the history of operation or fracture within one month were higher in PTE group. Though history of cardiovascular diseases differed between three groups, most subjects had well-controlled hypertension, only four subjects from the PTE group and six subjects from the CTEPH group were diagnosed with coronary artery disease (CAD). No subject had a history of cancer.

**Table 1:** Clinical data of subjects.

	CTEPH (n = 101)	PTE(n = 102)	Controls (n = 108)	p value
Age at first diagnosis, median [IQR] (years)	54.02±12.9 [13–85]	58.33±14.9 [18–84]	54.19±16.55 [18–86]	0.065
Male, n (%)	61 (60.4)	53 (52)	46 (42.6)	**0.036**
Smoke^*^, n (%)	16 (15.8)	16 (15.7)	17 (15.7)	1
Operation or fracture within 1 month, n (%)	0 (0)	22 (21.6)	0 (0)	**<0.001**
Cardiovascular disease history, n (%)	28 (27.7)	52 (51)	19 (17.6)	**<0.001**
Pulmonary arterial pressure (mmHg) [IQR] (n)	52±12.54 [27–89] (81)	<25	<25	
Pulmonary vascular resistance (dyn.sec/cm^5^) [IQR] (n)	1091.04±483.14 [242–3262] (75)	<100	<100	
Cardiac index (L/min/cm^2^) [IQR] (n)	3.5±1.14 [1.25–6.9] (75)	–	–	

Basic characteristic and risk factors for patients. Data are reported as number (%) or median (IQR, interquartile range); *P*-values were calculated by univariable analysis (x^2^-test).

Note: ^*^smoke : smoke≥300 cigarettes each year, and smoke within one month when enrolled.

### Genotype Polymorphism Frequencies and Allele Frequencies

Although polymorphisms are associated with CTEPH or PTE, no polymorphism was reported to be involved in CTEPH but not PTE. Since fibrin resistance has been suggested to be associated with CTEPH, polymorphisms possibly associated with fibrin resistance of the collected subjects were analyzed (Table 2 and Table S1). No significant deviation from Hardy-Weinberg equilibrium was observed in any polymorphism and group. The genotype frequencies of fibrinogen Bβ Arg448Lys, fibrinogen Bβ −148C/T, fibrinogen Bβ −455G/A, prothrombin 19911A/G, PAI-1 4G/5G, t-PA 7351C/T did not differ between the groups. Interestingly, differences of the genotype frequency (*P* = 0.034) and allele frequency (*P* = 0.01) of fibrinogen Aα Thr312Ala (A/G) were observed in different groups. There was significant difference in frequencies of genotype and allele gene between CTEPH subjects, controls (genotype frequencies: *P* = 0.017, allele frequencies: *P* = 0.005) and PTE subjects (genotype frequencies: *P* = 0.044, allele frequencies: *P* = 0.018), but there was no statistical difference between PTE subjects and controls in respect to frequencies of genotype and allele gene (genotype frequencies: *P* = 0.654, allele frequencies: *P* = 0.691). Logistic regression analysis confirmed that fibrinogen Aα Thr312Ala (A/G) increased the incidence of CTEPH compared with that of the controls (OR = 1.763 95%CI: 1.185–2.623) and PTE (OR = 1.623, 95%CI: 1.087–2.426) (Table 3).

**Table 2:** Genotype and allele frequencies of the Fibrinogen Aα Thr312Ala polymorphism.

Polymorphism	Genotype/allele	Frequency			p value	
		CTEPH	PTE	Control	Between 3 groups	Between 2 groups
**Aα Thr312Ala (A/G)** **rs6050**	A/A	25%(25/100)	37%(37/99)	43%(45/106)	***P*** ** = 0.034**	**CTEPH/control 0.017**
	A/G	57%(57/100)	55%(54/99)	48%(51/106)		PTE/control 0.654
	G/G	18%(18/100)	8%(8/99)	9%(10/106)		**CTEPH/PTE 0.044**
	**A**	55%(109/200)	64%(128/198)	67%(141/212)	***P*** ** = 0.01**	**CTEPH/control 0.005**
	**G**	45%(95/200)	35%(70/198)	33%(71/212)		PTE/control 0.691
						**CTEPH/PTE 0.018**

All frequencies are presented as % (n/total sample number). Genotype and allele frequencies were analyzed using the Chi-squared test, or Fisher’s exact test in the case of low numbers. CTEPH: chronic thromboembolic pulmonary hypertension; PTE: pulmonary thromboembolism. Thr: threonine; Ala alanine;

**Table 3:** Odd ratios and 95% CI of fibrinogen Aα Thr312Ala in CTEPH, PTE and controls.

Group	Adjusted *P* value	OR	Adjusted OR	Adjusted 95% CI
PTE/control	0.444	1.086	1.218	0.736–2.015
CTEPH/control	**0.004**	**1.763**	**2.037**	**1.262–3.289**
CTEPH/PTE	0.058	**1.623**	1.625	0.983–2.686

Odd ratios (OR) and 95% confidence interval (95%CI) of fibrinogen Aα Thr312Ala in CTEPH, PTE and controls. OR and 95%CI of fibrinogen Aα Thr312Ala to CTEPH and PTE before and after adjustment of age, sex, smoke, operation or fracture history within 1 month and cardiovascular disease history. All OR and 95%CI are calculated by multiple logistic regression models.

### Fibrinogen Aα Thr312Ala (A/G) is an Independent Risk Factor for CTEPH, but not PTE

The effects of fibrinogen Aα Thr312Ala (A/G) on CTEPH and PTE were further analyzed with adjustment of the demographic differences between the groups (Table 3). The adjusted OR (2.037) and 95% CI (1.262–3.289) showed that fibrinogen Aα Thr312Ala (A/G) was an independent risk factor of CTEPH but not PTE after adjustments of age, sex, smoking status, operation or fracture history within 1 month and cardiovascular disease history in multiple logistic regression analysis (Table 3).

### Fibrin Derived from Subjects with CTEPH is More Resistant to Lysis than that of PTE or Controls

**Figure 1:**
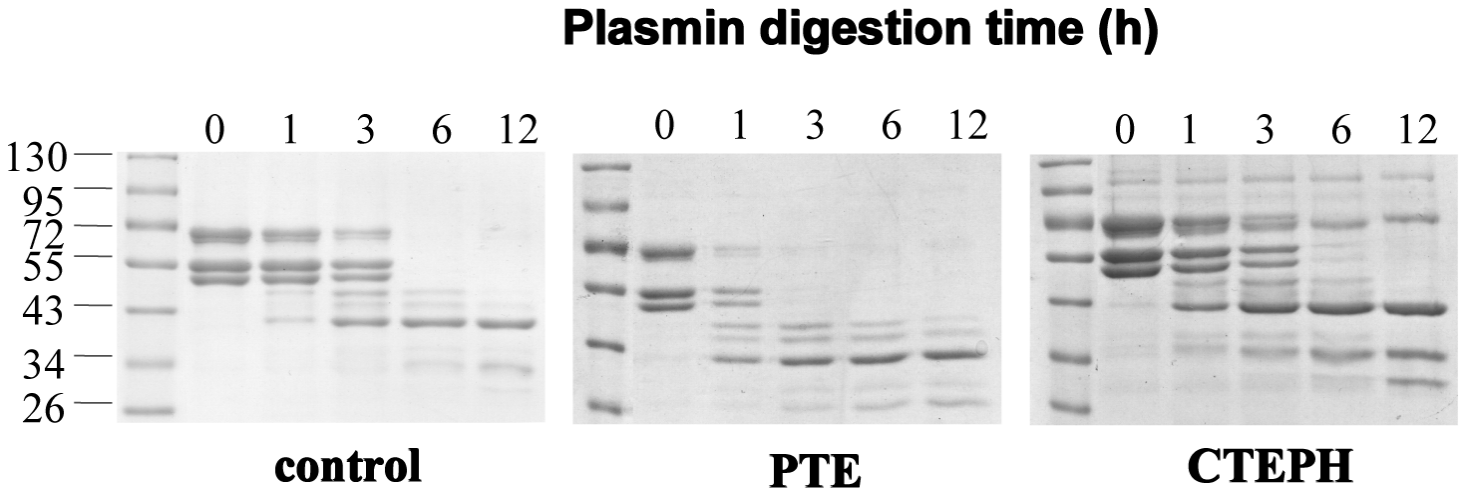
Plasmin-mediated cleavage of fibrin polymers from human subjects. Fibrin polymers from control (left), PTE (middle) and CTEPH (right) subjects were analyzed for fibrin cleavage following plasmin treatment at the indicated time points. Samples were resolved by 10% SDS-PAGE and proteins indicated by Coomassie blue staining. The bands near 72 kD, 55 kD, and just below the 55 kD marker represent alpha, beta, and gamma chains of fibrin, respectively.

**Figure 2:**
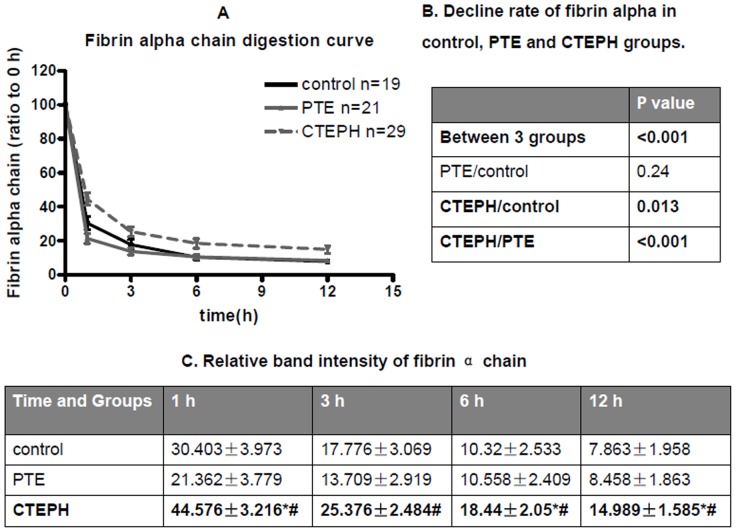
Fibrin α chain digestion curve of control, PTE, and CTEPH groups. (A) The band intensity of the stained gels was quantified by densitometry with Image J software and the corresponding data plotted as a function of time. The black solid line, gray solid line and gray dash line represent control, PTE, and CTEPH subjects, respectively. (B) Decline rate of fibrin alpha in control, PTE and CTEPH groups. *P* value of fibrin alpha digestion curves between control, PTE and CTEPH. *P* values are calculated by repeated measurement of categorical data. (C) Relative band intensity of fibrin α chain in different time of three groups. Data are presented as ratio of band intensity to time at 0 h and are reported as mean±SEM. *P* value was calculated v.s. control (*) or PTE (**#**) by repeated measurement of categorical data.

Plasmin-mediated fibrin degradation from CTEPH subjects, PTE subjects and controls was compared. The decline of fibrin over time was calculated, which was significantly decreased in CTEPH subjects in comparison with controls and PTE subjects, implying that fibrin derived from CTEPH subjects is much resistant to lysis (Fig. 1 and Fig. 2). However, there was no significant difference between PTE subjects and controls (Fig.1 and Fig. 2). In one hour to twelve hours, the fibrin alpha chain band intensity decreased in different degrees from different groups (Fig. 2). Similar results were observed in fibrin beta chain and fibrin gamma chain from different groups (data not shown).

### Fibrin Resistance and Fibrinogen Aα Ala312Ala (G/G) Genotype

**Figure 3:**
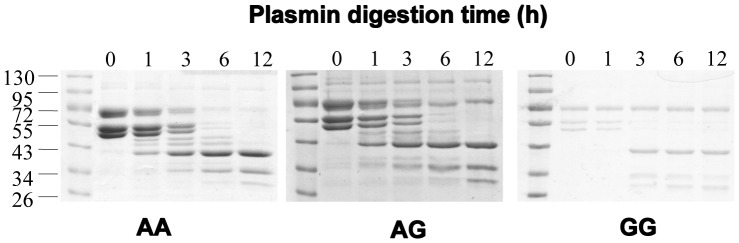
Plasmin-mediated cleavage of fibrin polymers of different genotypes from CTEPH subjects. Fibrin polymers from A/A (left), A/G (middle) and G/G (right) Aα Thr312Thr genotypes were analyzed for fibrin cleavage following plasmin treatment at the indicated time points. Samples were resolved by 10% SDS-PAGE and proteins indicated by Coomassie blue staining. The bands near 72 kD, 55 kD, and just below the 55 kD marker represent alpha, beta, and gamma chains of fibrin, respectively.

**Figure 4:**
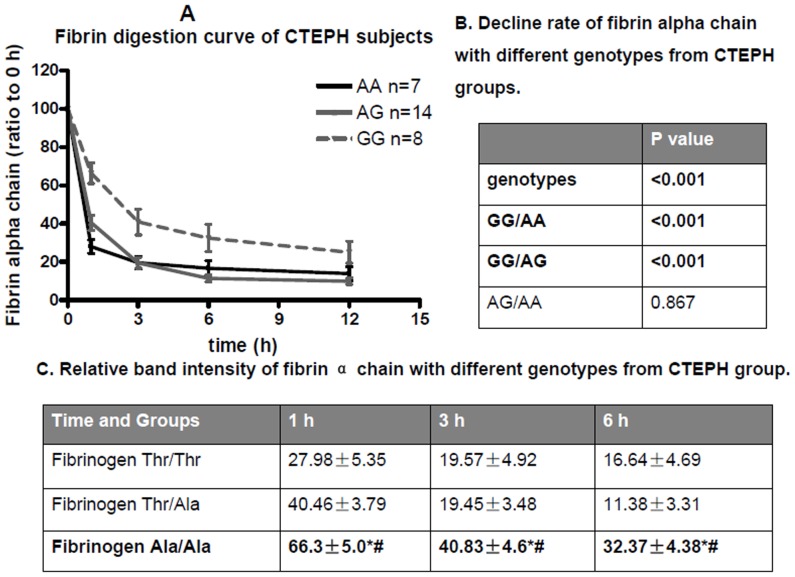
Fibrin α chain digestion curve of different genotypes from CTEPH patients. (A) The band intensity of the stained gels was quantified by densitometry with Image J software and the corresponding data plotted as a function of time. The black solid line, gray solid line and gray dash line represent A/A, A/G, and G/G genotypes, respectively. (B) Decline rate of fibrin alpha chain with different genotypes from CTEPH groups. *P* value of fibrin alpha digestion curves between different genotypes of fibrinogen Aα Thr312Ala in CTEPH groups. *P* values are calculated by repeated measurement of categorical data. GG: fibrinogen Aα Thr312Ala (GG) genotype; GA: fibrinogen Aα Thr312Ala (GA) genotype; AA: fibrinogen Aα Thr312Ala (AA) genotype. (C) Relative band intensity of fibrin α chain with different genotypes from CTEPH group. Data are presented as ratio of band intensity to time at 0 h and are reported as mean ± SEM. *P* value was calculated v.s. control (*) or PTE (**#**) by repeated measurement of categorical data.

The relationship between fibrin degradation and fibrinogen Aα Thr312Ala (A/G) genotypes in CTEPH subjects was also studied, and results demonstrated that there were differences between different fibrinogen Aα Thr312Ala (A/G) genotypes and fibrin resistance (Fig. 3 and Fig. 4). In one hour to twelve hours, the fibrin alpha chain band intensity decreased in different degree in different groups (Fig. 4).

## Discussion

For the first time, we report a specific polymorphism responsible for CTEPH but not PTE in Chinese patients. Our data shows that fibrinogen Aα Thr312Ala polymorphism is an independent risk factor of CTEPH due to fibrin resistance to lysis by plasmin and the fact that it is related to the fibrinogen Aα Thr312Ala genotypes in CTEPH patients.

Indeed, CTEPH is usually considered as a consequence of acute PTE [7], [16], and a recent study showed that CTEPH generally develops after acute PTE within a shorter period [17]. Therefore, closer monitoring of high-risk cases is particularly important in early diagnosis and treatment.

Previously, a case control study with 214 CTEPH subjects found that fibrinogen Aα Thr312Ala polymorphism is associated with CTEPH, but the study did not include a group of PTE subjects [13]. In total, 45 subjects with acute PTE enrolled before 2008 have been followed up, and we excluded 1 subject when CTEPH occurred. This CTEPH incidence from acute PTE is consistent with the previous report of 0.5–3.8% [18], [19]. Interestingly, we found that fibrinogen Aα Thr312Ala polymorphism is associated with CTEPH but not PTE as an independent risk factor (Table 3). This study also suggested a potential difference of fibrinogen Aα Thr312Ala polymorphism between CTEPH and PTE (*P* = 0.058) (Table 3).

Genotypes and allele frequencies of other previously reported polymorphisms that associated with thrombotic diseases [fibrinogen Bβ Arg448Lys (rs420), fibrinogen Bβ −148C/T (rs1800787), fibrinogen Bβ −455G/A (rs1800790), prothrombin 19911A/G (rs3136516), plasminogen activator inhibitor-1 (PAI-1 4G/5G, rs1799768), tissue plasminogen activator 7351C/T (t-PA, rs2020918)] did not show a difference between CTEPH and control/PTE [20 21]. Nonetheless, our results are in line with a large cohort study, which suggested only fibrinogen Aα Thr312Ala polymorphism is associated with CTEPH among the common polymorphisms in haemostatic or fibrolysis system [13].

Since increased fibrinogen levels have been reported to be associated with risk for venous thrombosis [22], we investigated whether fibrinogen Aα Thr312Ala (A/G) was associated with plasma levels of fibrinogen in these three groups. However, there is no obvious difference of fibrinogen levels between three genotypes, and after adjustment of age, sex, smoking status, operation or fracture history within 1 month and cardiovascular disease history in multiple logistic regression analysis, the difference is not significant (data not shown). This result is in accordance with previous report [23] which also showed that fibrinogen Aα Thr312Ala (A/G) was not associated with fibrinogen level. Linkage disequilibrium (LD) has been shown for fibrinogen Aα Thr312Ala (A/G) polymorphism and the haplotype of the fibrinogen gamma gene, FGG-H2, which associates with reduced fibrinogen gamma prime levels (γ’). Further, it has been reported that reduced fibrinogen γ’ is an independent risk factor for venous thrombosis [23], [24], so this aspect maybe one of the mechanisms of fibrinogen Aα Thr312Ala (A/G) polymorphism to CTEPH. But as PTE also belongs to venous thrombosis, this hypothesis can not fully explain the mechanism of fibrinogen Thr312Ala (A/G) polymorphism.

Fibrin clots of patients with CTEPH were much resistant to fibrolysis than that of healthy control subjects [9]. Fibrin derived from subjects with CTEPH and pulmonary hypertension other than thromboembolic [10] was reported to be resistant to lysis. Fibrin resistance occurred in a smaller extent in subjects with prior PTE with no hypertension [10]. Our data show fibrin from CTEPH subjects was more resistant to lysis as compared to both healthy controls and PTE subjects without pulmonary hypertension (Figure 1 and 2). The latter conclusion is slightly different from that of the previous report [25]. The reason might be that the diversiform influencing factors of fibrin resistance to lysis [25] such as post-translational modifications [26] other than genetic polymorphisms.

Genetic mutation and polymorphisms contribute to the structure of the fibrin clot[27]–[29]. Ala312 fibrin clots have thicker fibers and more extensive α–chain cross-linking than that of Thr312 [15]. It is possible that the substitution of threonine to alanine at position 312 of the C-terminal α-chain enhances lateral aggregation leading to thicker fibers, consistent with the role of the αC domains in lateral aggregation [30]. We tried to find the possible relationship between the fibrin alpha chain degradation rate and the genotypes of fibrinogen FGA (A gene that encodes for the fibrinogen alpha chain) Thr312Ala (A/G) polymorphism in CTEPH subjects. We found that fibrin with FGA GG genotype (which encode fibrinogen with Ala312Ala) is more resistant than the fibrin with FGA AG genotype (which encode fibrinogen with Thr312Ala) or FGA AA genotype (which encode fibrinogen with Thr312Thr) genotype. The low frequency of FGA GG genotypes and some limitations in sample collection did not allow us to properly assess the degradation rate of the fibrin alpha chain between different genotypes of FGA Thr312Ala (A/G) polymorphism from the PTE subjects and controls. However, we compared the lysis curve of CTEPH with fibrinogen Thr312Ala AG and AA genotypes with the total lysis curve of PTE subjects (with fibrinogen Thr312Ala AG and AA genotypes) or controls (with fibrinogen Thr312Ala AG and AA genotypes) and found no significant difference (*P* = 0.086), further supporting the hypotheses that the resistance of fibrin in CTEPH patients is mainly determined by GG genotype.

Our study suggests that fibrinogen Aα Thr312Ala polymorphism is a potential biomarker in identifying CTEPH from acute PTE. However, the sample size in this study may limit our power in identifying other factors that influence the fibrin structure and its resistance to lysis [31]. Further *in vitro* and *in vivo* functional assessments are needed to determine the genetic effect of Thr312Ala polymorphism on fibrinogen structure and function. Our results demonstrate that fibrinogen Aα Thr312Ala polymorphism may contribute to chronic thromboembolic pulmonary hypertension through increasing fibrin resistance, implying that PTE subjects with fibrinogen Aα Ala312Ala genotype in clinic may need long-term anticoagulation therapy.

## Supporting Information

Table S1 **Genotype and allele polymorphism frequencies.** Genotype and allele frequencies of the polymorphisms detected. All frequencies are presented as % (n/total sample number). Any SNP deviated significantly from Hardy-Weinberg equilibrium was excluded for statistic analysis. Genotype and allele frequencies were analyzed using the Chi-squared test, or Fisher’s exact test in the case of low numbers. CTEPH: chronic thromboembolic pulmonary hypertension; PTE: pulmonary thromboembolism. t-PA: tissue plasminogen activator; PAI: plasminogen activator inhibitor; Thr: threonine; Ala alanine; Arg: arginine; Lys: lysine.
(DOC)

Protocol S1 **Method of fibrinogen purification by ethanol precipitation.** Fibrinogen was purified using an ethanol precipitation method as follows: Place 1 ml plasma in ice water bath, using a plastic stick stir in 0.215 ml solution “A” [1 ml 95% EtOH+1 ml 55 mM NaCit (sodium citrate) pH7.0]. Adding the solution in a drop wise fashion while cooling to −3°C. Let stand for 15 minutes. Centrifuge at −3°C at 3500 rpm for 20 minutes. Pipette off supernatant and discard. Add 0.55 ml Solution “B” (480 µl 95% EtOH +5.52 ml 0.055 M NaCit pH6.4) and stir for 10 minutes at −3°C as the protein sticks to the plastic stick at this step. Centrifuge at −3°C at 3500 rpm for 10 minutes. Pipette off supernatant and discard. Add 0.275 ml Solution “C” (0.055 M NaCit pH6.4) at room temperature to dissolve precipitate fully by gently shaking tube for about 20 minutes, try not to make bubbles. Place tube in ice water bath, add 0.0475 ml Solution “D” (96 µl Absolute EtOH +632 µl NaCit pH6.4). Centrifuge at −3°C at 3500 rpm for 10 minutes. Remove supernatant and save it, this contains the fibrinogen, discard the pellet. Add 0.14 ml Solution “E” (283 µl Absolute EtOH +1 ml 0.055 M NaCit pH6.4) drop wise to supernatant while stirring at −3°C, then let sit for 10 minutes at −3°C. Centrifuge at −3°C at 3500 rpm for 15 minutes. Discard supernatant. Re-dissolve in 0.25 ml Solution “F” (20 mM NaCit, 150 mM NaCl, pH 7.0) and make sure all the small particles are dissolved.
(DOC)
