# Supplementary material for: Fibrinogen Aα Thr312Ala Polymorphism Specifically Contributes to Chronic Thromboembolic Pulmonary Hypertension by Increasing Fibrin Resistance
Source: PLoS One. 2013 Jul 22;8(7):e69635. doi: 10.1371/journal.pone.0069635 (PMC3718692; doi:10.1371/journal.pone.0069635)
Supplement: Table S1 — Genotype and allele polymorphism frequencies. Genotype and allele frequencies of the polymorphisms detected. All frequencies are presented as % (n/total sample number). Any SNP deviated significantly from Hardy-Weinberg equilibrium was excluded for statistic analysis. Genotype and allele frequencies were analyzed using the Chi-squared test, or Fisher’s exact test in the case of low numbers. CTEPH: chronic thromboembolic pulmonary hypertension; PTE: pulmonary thromboembolism. t-PA: tissue plasminogen activator; PAI: plasminogen activator inhibitor; Thr: threonine; Ala alanine; Arg: arginine; Lys: lysine. (DOC) [file pone.0069635.s001.doc]

**Table S1. Genotype and allele polymorphism frequencies**.

| Polymorphisms | Genotype/allele | Frequency | | | p value | |
| --- | --- | --- | --- | --- | --- | --- |
|  |  | CTEPH | PTE | Control | Between 3 groups | Between 2 groups |
| **Aα Thr312Ala (A/G)**  **rs6050** | A/A | 25%(25/100) | 37%(37/99) | 43%(45/106) | ***P* = 0.034** | **CTEPH/control 0.017** |
|  | A/G | 57%(57/100) | 55%(54/99) | 48%(51/106) |  | PTE/control 0.654 |
|  | G/G | 18%(18/100) | 8%(8/99) | 9%(10/106) |  | **CTEPH/PTE 0.044** |
|  | **A** | 55%(109/200) | 64%(128/198) | 67%(141/212) | ***P* = 0.01** | **CTEPH/control 0.005** |
|  | **G** | 45%(95/200) | 35%(70/198) | 33%(71/212) |  | PTE/control 0.691 |
|  |  |  |  |  |  | **CTEPH/PTE 0.018** |
| **Bβ Arg448Lys (G/A)**  **rs4220** | G/G | 77%(78/101) | 69%(70/101) | 66%(71/108) | *P* = 0.327 | CTEPH/control 0.112 |
|  | G/A | 21%(21/101) | 30%(30/101) | 33%(36/108) |  | PTE/control 0.853 |
|  | A/A | 2%(2/101) | 1%(1/101) | 1%(1/108) |  | CTEPH/PTE 0.308 |
|  | G | 88%(177/202) | 83%(170/202) | 82%(178/216) | *P* = 0.324 | CTEPH/control 0.136 |
|  | A | 12%(25/202) | 17%(32/202) | 18%(38/216) |  | PTE/control 0.632 |
|  |  |  |  |  |  | CTEPH/PTE 0.317 |
| **Bβ-148 C/T**  **rs1800787** | C/C | 76%(74/97) | 68%(68/100) | 65%(70/107) | *P* = 0.376 | CTEPH/control 0.202 |
|  | C/T | 22%(21/97) | 30%(30/100) | 30%(32/107) |  | PTE/control 0.564 |
|  | T/T | 2%(2/97) | 2%(2/100) | 5%(5/107) |  | CTEPH/PTE 0.407 |
|  | C | 87%(169/194) | 83%(166/200) | 80%(172/214) | *P* = 0.186 | CTEPH/control 0.066 |
|  | T | 13%(25/194) | 17%(34/200) | 20%(42/214) |  | PTE/control 0.49 |
|  |  |  |  |  |  | CTEPH/PTE 0.253 |
| **Bβ-455 G/A**  **rs1800790** | G/G | 54%(52/97) | 70%(71/102) | 65%(70/107) | *P* = 0.204 | CTEPH/control 0.221 |
|  | G/A | 43%(42/97) | 28%(29/102) | 32%(34/107) |  | PTE/control 0.785 |
|  | A/A | 3%(3/97) | 2%(2/102) | 3%(3/107) |  | CTEPH/PTE 0.067 |
|  | G | 75%(146/194) | 84%(171/204) | 81%(174/214) | *P* = 0.875 | CTEPH/control 0.962 |
|  | A | 25%(48/194) | 16%(33/204) | 19%(40/214) |  | PTE/control 0.636 |
|  |  |  |  |  |  | CTEPH/PTE 0.673 |
| **prothrombin 19911**  **A/G rs3136516** | GG | 68%(65/96) | 77%(75/98) | 72%(74/103) | *P* = 0.296 | CTEPH/control 0.19 |
|  | GA | 26%(26/96) | 21%(21/98) | 27%(28/103) |  | PTE/control 0.542 |
|  | **AA** | 5%(5/96) | 2%(2/98) | 1%(1/103) |  | CTEPH/PTE 0.279 |
|  | **G** | 81%(156/192) | 87%(171/196) | 85%(176/206) | *P* = 0.243 | CTEPH/control 0.262 |
|  | **A** | 19%(36/192) | 13%(25/196) | 15%(30/206) |  | PTE/control 0.598 |
|  |  |  |  |  |  | CTEPH/PTE 0.105 |
| **t-PA -7351C/T**  **rs2020918** |  | All alleles are CC (101) | All alleles are CC (98) | All alleles are CC (104) | *P* = 1 | *P* = 1 |
| **PAI-1 675 4G/5G**  **rs1799768** | GG | 20%(20/99) | 16%(16/98) | 20%(21/103) | *P* = 0.949 | CTEPH/control 0.999 |
|  | **G** | 48%(47/99) | 51%(50/98) | 48%(49/103) |  | PTE/control 0.749 |
|  | Del | 32%(32/99) | 33%(32/98) | 32%(33/103) |  | CTEPH/PTE 0.766 |
|  | G | 44%(87/198) | 42%(82/196) | 44%(91/206) | *P* = 0.875 | CTEPH/control 0.962 |
|  | Del | 56%(111/198) | 58%(114/196) | 56%(115/206) |  | PTE/control 0.636 |
|  |  |  |  |  |  | CTEPH/PTE 0.673 |

**Table S1. Genotype and allele frequencies of the polymorphisms detected.** All frequencies are presented as % (n/total sample number). Any SNP deviated significantly from Hardy-Weinberg equilibrium was excluded for statistic analysis. Genotype and allele frequencies were analyzed using the Chi-squared test, or Fisher’s exact test in the case of low numbers. CTEPH: chronic thromboembolic pulmonary hypertension; PTE: pulmonary thromboembolism. t-PA: tissue plasminogen activator; PAI: plasminogen activator inhibitor; Thr: threonine; Ala alanine; Arg: arginine; Lys: lysine.
