# Supplementary material for: Fibrinogen Aα Thr312Ala Polymorphism Specifically Contributes to Chronic Thromboembolic Pulmonary Hypertension by Increasing Fibrin Resistance
Source: PLoS One. 2013 Jul 22;8(7):e69635. doi: 10.1371/journal.pone.0069635 (PMC3718692; doi:10.1371/journal.pone.0069635)
Supplement: Protocol S1 — Method of fibrinogen purification by ethanol precipitation. Fibrinogen was purified using an ethanol precipitation method as follows: Place 1 ml plasma in ice water bath, using a plastic stick stir in 0.215 ml solution “A” [1 ml 95% EtOH+1 ml 55 mM NaCit (sodium citrate) pH7.0]. Adding the solution in a drop wise fashion while cooling to −3°C. Let stand for 15 minutes. Centrifuge at −3°C at 3500 rpm for 20 minutes. Pipette off supernatant and discard. Add 0.55 ml Solution “B” (480 µl 95% EtOH +5.52 ml 0.055 M NaCit pH6.4) and stir for 10 minutes at −3°C as the protein sticks to the plastic stick at this step. Centrifuge at −3°C at 3500 rpm for 10 minutes. Pipette off supernatant and discard. Add 0.275 ml Solution “C” (0.055 M NaCit pH6.4) at room temperature to dissolve precipitate fully by gently shaking tube for about 20 minutes, try not to make bubbles. Place tube in ice water bath, add 0.0475 ml Solution “D” (96 µl Absolute EtOH +632 µl NaCit pH6.4). Centrifuge at −3°C at 3500 rpm for 10 minutes. Remove supernatant and save it, this contains the fibrinogen, discard the pellet. Add 0.14 ml Solution “E” (283 µl Absolute EtOH +1 ml 0.055 M NaCit pH6.4) drop wise to supernatant while stirring at −3°C, then let sit for 10 minutes at −3°C. Centrifuge at −3°C at 3500 rpm for 15 minutes. Discard supernatant. Re-dissolve in 0.25 ml Solution “F” (20 mM NaCit, 150 mM NaCl, pH 7.0) and make sure all the small particles are dissolved. (DOC) [file pone.0069635.s002.doc]

Protocol S1: Method of fibrinogen purification by ethanol precipitation

Fibrinogen was purified using an ethanol precipitation method as follows: Place 1 ml plasma in ice water bath, using a plastic stick stir in 0.215 ml solution “A” [1 ml 95% EtOH+ 1 ml 55 mM NaCit (sodium citrate) pH7.0]. Adding the solution in a drop wise fashion while cooling to -3 ºC. Let stand for 15 minutes. Centrifuge at -3 ºC at 3500 rpm for 20 minutes. Pipette off supernatant and discard. Add 0.55 ml Solution “B” (480 μl 95% EtOH + 5.52 ml 0.055 M NaCit pH6.4) and stir for 10 minutes at -3 ºC as the protein sticks to the plastic stick at this step. Centrifuge at -3 ºC at 3500 rpm for 10 minutes. Pipette off supernatant and discard. Add 0.275 ml Solution “C” (0.055 M NaCit pH6.4) at room temperature to dissolve precipitate fully by gently shaking tube for about 20 minutes, try not to make bubbles. Place tube in ice water bath, add 0.0475 ml Solution “D” (96 μl Absolute EtOH + 632 μl NaCit pH6.4). Centrifuge at -3 ºC at 3500 rpm for 10 minutes. Remove supernatant and save it, this contains the fibrinogen, discard the pellet. Add 0.14 ml Solution “E” (283 μl Absolute EtOH + 1 ml 0.055 M NaCit pH6.4) drop wise to supernatant while stirring at -3 ºC, then let sit for 10 minutes at -3 ºC. Centrifuge at -3 ºC at 3500 rpm for 15 minutes. Discard supernatant. Re-dissolve in 0.25 ml Solution “F” (20 mM NaCit, 150 mM NaCl, pH 7.0) and make sure all the small particles are dissolved.
